# LPS Increases MUC5AC by TACE/TGF-**α**/EGFR Pathway in Human Intrahepatic Biliary Epithelial Cell

**DOI:** 10.1155/2013/165715

**Published:** 2013-08-20

**Authors:** Zipei Liu, Feng Tian, Xiaobin Feng, Yu He, Peng Jiang, Jianwei Li, Fei Guo, Xin Zhao, Hong Chang, Shuguang Wang

**Affiliations:** ^1^Institute of Hepatobiliary Surgery, Southwest Hospital, Third Military Medical University, No. 29 Gaotanyan Street, Shapingba District, Chongqing 400038, China; ^2^The 306th Hospital of PLA, No. 9 Anxiang Road (N), Chaoyang District, Beijing 100101, China

## Abstract

*Background*. Mucin 5AC (MUC5AC) overproduction plays important roles in stone formation and recurrence of hepatolithiasis. We aim to investigate the involved mechanism and the potential target to block this process. *Methods*. 42 bile duct samples from hepatolithiasis and 15 normal bile duct samples from hemangioma patients were collected for detecting MUC5AC expression by immunohistochemistry. MUC5AC and phosphoepidermal growth factor receptor (pEGFR) expressions in human intrahepatic biliary epithelial cells (HIBECs) cultured with or without lipopolysaccharide (LPS) were detected by real-time PCR and western blot analysis. Transforming growth factor-**α** (TGF-**α**) secretion in HIBECs was detected by ELISA. *Results*. MUC5AC was overexpressed in bile ducts of hepatolithiasis samples compared with bile ducts from hemangioma samples. LPS upregulated MUC5AC expression in HIBECs. LPS promoted EGFR activation, and inhibiting EGFR activation by AG1478 significantly decreased LPS-induced MUC5AC overexpression in HIBECs. Moreover, LPS increased TGF-**α** secretion, and inhibiting tumor necrosis factor-**α** converting enzyme (TACE), which has been implicated in ectodomain cleavage of TGF-**α**, significantly inhibited LPS-induced EGFR activation and subsequent MUC5AC overexpression in HIBECs. *Conclusion*. Our results suggested that LPS increases MUC5AC expression through the TACE/TGF-**α**/EGFR pathway in HIBECs. This new finding might give light to the prevention of stone formation and recurrence of hepatolithiasis.

## 1. Introduction

Hepatolithiasis is a prevalent disease in Asian regions, particularly in China [1]. Despite the therapeutic effects of surgical and nonsurgical procedures [2, 3], stone recurrence is observed in most hepatolithiasis patients, resulting in serious complications, such as cholangitis, biliary stenosis, biliary fibrosis, and even cholangiocarcinoma, which always necessitates reoperation and significantly limits long-term survival [4]. Therefore, investigating the mechanism underlying stone formation and identifying potential targets to prevent stone recurrence remains a priority.

Although hepatolithiasis is a multifactorial disease, bacteria infection and bile flow retardation form a vicious cycle which promotes stone formation and recurrence, and mucin overexpression plays important roles in this process [5, 6]. Mucins are the primary components of mucus which is the main defence barrier against damage factors in the gastrointestinal tract, including the bile duct [7]. During hepatolithiasis development, lipopolysaccharide (LPS) increases mucin 5AC (MUC5AC) expression, resulting in the increased mucosity and bile flow retardation, which promotes stone formation and recurrence [8, 9]. Therefore, investigating the regulatory mechanism of MUC5AC overexpression and finding potential targets to inhibit this process might be a strategy for preventing stone recurrence and will improve the long-term survival of hepatolithiasis.

MUC5AC is regulated by different pathways in different cell types. It has been reported that epidermal growth factor receptor (EGFR) activation plays important roles in regulation of MUC5AC expression in bronchial epithelial cells [10]. Meanwhile, EGFR activation is associated with MUC5AC overexpression in gallbladder epithelial cells which promotes stone formation in cholecystolithiasis [11]. However, because of the differences between cholecystolithiasis and hepatolithiasis and the heterogeneity between gallbladder and intrahepatic epithelial cells, whether EGFR activation is involved in MUC5AC overproduction in intrahepatic biliary epithelial cells needs to be investigated further. In addition, among the several ligands of EGFR, transforming growth factor-*α* (TGF-*α*), a potent ligand for EGFR, is reported to be involved in regulation of MUC5AC expression in human pulmonary mucoepidermoid carcinoma cells [12]. Based on the above reports, we postulate that TGF-*α*/EGFR pathway might be involved in regulating MUC5AC overproduction in intrahepatic biliary epithelial cells. Besides, tumour necrosis factor-*α* converting enzyme (TACE) has been implicated in the ectodomain cleavage of TGF-*α* which is important for TGF-*α* maturation and secretion. It has been reported that TACE activity is essential for TGF-*α* maturation in many inflammatory diseases [13]. However, whether TACE is also involved in MUC5AC overproduction in biliary epithelial cells remains unclear.

In the present study, we firstly validated the MUC5AC expression in clinical hepatolithiasis samples. As a previous study has showed that LPS induced MUC5AC overexpression in cultured biliary epithelial cells [8], we then investigated the mechanism of regulating MUC5AC expression in human intrahepatic biliary epithelial cells (HIBECs).

## 2. Materials and Methods

### 2.1. Clinical Samples

A total of 42 bile duct samples obtained from hepatolithiasis patients and 15 normal bile duct samples obtained from haemangioma patients undergoing surgical resection at the Southwest Hospital in 2012 were collected according to the protocols of the Institutional Review Board of the Southwest Hospital, Third Military Medical University.

### 2.2. Immunohistochemistry (IHC)

The expression of MUC5AC and phospho-EGFR (pEGFR) in the paraffin-embedded clinical samples was detected using IHC, as previously described [14]. Paraffin-embedded sections were dewaxed in xylene and rehydrated in a series of graded alcohol. Antigen retrieval was performed after the elimination of endogenous peroxidase activity with a 3% H_2_O_2_ in methanol. Sections were blocked with normal serum and incubated with primary antibodies (Muc5AC, Santa Cruz, 1 : 200; pEGFR, Biosynthesis Biotechnology, 1 : 200) at 4°C overnight. Negative controls were performed by replacing primary antibodies with PBS buffer. Subsequently, sections were incubated with horseradish peroxidase labeled anti-mouse or anti-rabbit IgG (ZSGB-BIO, Beijing, China) for 30 min at room temperature. Finally, sections were counterstained with hematoxylin and visualized with 3,3-diaminobenzidine tetrahydrochloride (ZSGB-BIO, Beijing, China). Two independent investigators assessed the subcellular localization and percentage of stained cells for each sample.

MUC5AC and pEGFR expressions were evaluated as previous studies with minor modification [15, 16]. In general, MUC5AC showed cytoplasmic staining in the epithelial cells of bile ducts and peribiliary glands. Greater than 10% epithelial cells with cytoplasmic staining was considered as a positive result. The pEGFR showed cytoplasmic staining in the epithelial cells of bile ducts and peribiliary glands. Greater than 10% epithelial cells with membrane staining was considered as a positive result.

### 2.3. Cell Culture and Reagents

HIBECs were purchased from the ScienCell Research Laboratories (San Diego, CA, USA). The HIBECs were cultured in biliary epithelial cell culture media comprising DMEM/F12 (1 : 1) supplemented with 10% foetal bovine serum (FBS), 25 ng/mL epidermal growth factor, 393 ng/mL dexamethasone, 100 IU/mL penicillin, and 100 *μ*g/mL streptomycin at 37°C in a 5% CO_2_-humidified atmosphere. The HIBECs were treated with 100 *μ*g/mL of LPS (Sigma) for 24 h as previously described [17]. The cells were treated with 10 *μ*g/mL of TAPI-1 (Calbiochem) or AG1478 (Sigma) to block TACE activity or EGFR phosphorylation, respectively.

### 2.4. Real-Time PCR

Total RNA was extracted using RNAiso Plus (Takara, China). The cDNA synthesis was performed according to the manufacturer's instructions (SYBR ExScript RT-PCR Kit, TaKaRa, China). Quantitative PCR was performed using the SYBR Premix Ex Taq II Kit (TaKaRa, China) with a LightCycler System. The PCR reactions for all assays were performed at 94°C for 30 seconds, followed by 40 amplification cycles at 94°C for 5 seconds, 61°C for 30 seconds, and 72°C for 30 seconds. GAPDH mRNA was used to normalise the RNA input. The primer sequences used in these studies are shown in Table 1.

### 2.5. Western Blot Analysis

Total proteins were isolated using RIPA Lysis Buffer (Beyotime, China). For immunoblotting, equal amounts of proteins were separated through 5%–12% SDS-PAGE and electrophoretically transferred onto nitrocellulose membranes (Millipore). The membranes were blocked in TBST containing 5% milk for 2 hours at RT and blotted with the appropriate primary antibody overnight at 4°C: anti-MUC5AC (1 : 500, Santa Cruz Biotechnology), anti-pEGFR (1 : 500, Santa Cruz Biotechnology), and anti-GAPDH (1 : 500, Biotechnology). After washing with TBST and subsequent incubation with either anti-rabbit or anti-mouse horseradish peroxidase-conjugated secondary antibodies (Biosynthesis Biotechnology, China) for 2 h at room temperature, the immunocomplexes were visualised using chemiluminescence (GE, USA) according to the manufacturer's protocol. Specific protein bands were photographed, and immunoquantification was accomplished through densitometric analysis using Quantity One software (Bio-Rad, Hercules, CA, USA).

### 2.6. Enzyme-Linked Immunosorbent Assay (ELISA)

The supernatants of cultured biliary epithelial cells were collected. The TGF-*α* concentration was detected using the TGF-*α* ELISA Kit (Westang Biotechnology, Shanghai) according to the manufacturer's protocol. 

### 2.7. Statistical Analysis

Data was analyzed using SPSS 17.0. Continuous data was measured by *t*-test. For categorical data, chi-square analysis or fisher's exact test was used. Statistical significance was set at *P* < 0.05.

## 3. Results

### 3.1. MUC5AC Was Overexpressed in the Bile Ducts of Hepatolithiasis Patients

**Figure 1:**
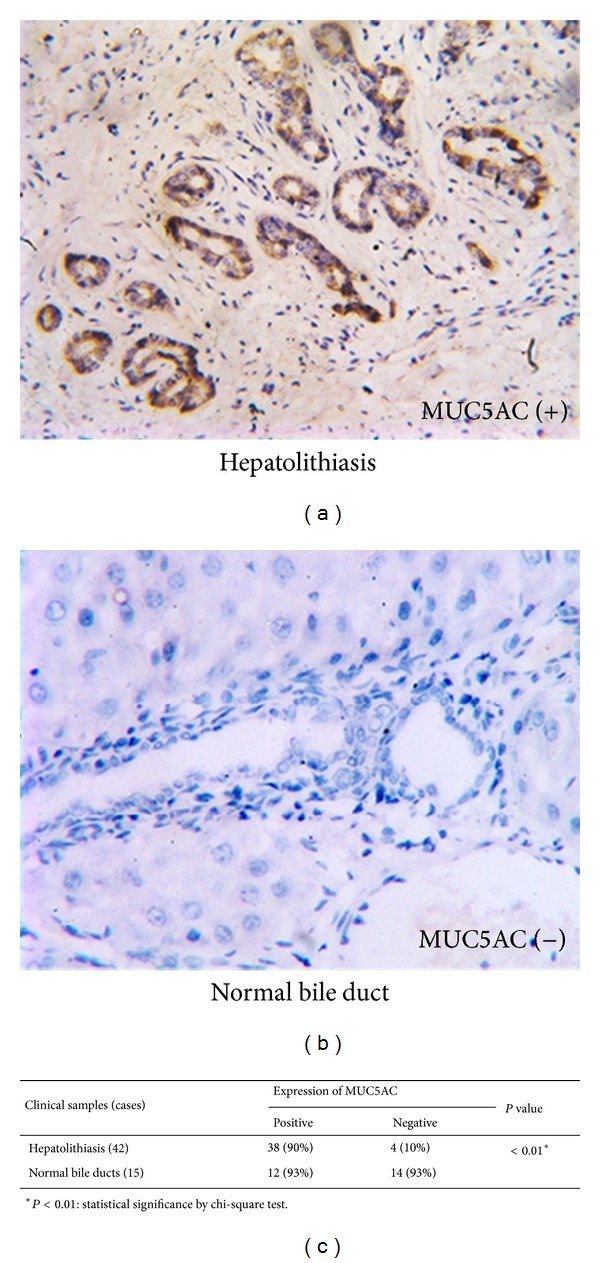
MUC5AC expression in clinical samples by IHC. (a) Positive staining of MUC5AC in bile duct samples obtained from hepatolithiasis patients. (b) Negative MUC5AC staining in normal bile duct samples obtained from haemangioma patients. (c) MUC5AC was overexpressed in bile duct samples obtained from hepatolithiasis patients.

The IHC analysis revealed that 38 of the 42 (90%) bile duct samples obtained from hepatolithiasis patients showed positive MUC5AC staining (Figure 1(a)). MUC5AC was primarily expressed in the cytoplasm of biliary epithelial cells. However, only 1 of the 15 normal bile duct tissue samples (7%) showed MUC5AC-positive staining, and 14 samples (93%) showed MUC5AC-negative staining (Figure 1(b)). The rate of MUC5AC positive staining in normal bile duct samples was significantly lower than that in the hepatolithiasis samples (*P* < 0.01, Figure 1(c)). These data showed that MUC5AC was overexpressed in the bile ducts of hepatolithiasis patients.

### 3.2. LPS Increased MUC5AC Expression in HIBECs

**Figure 2:**
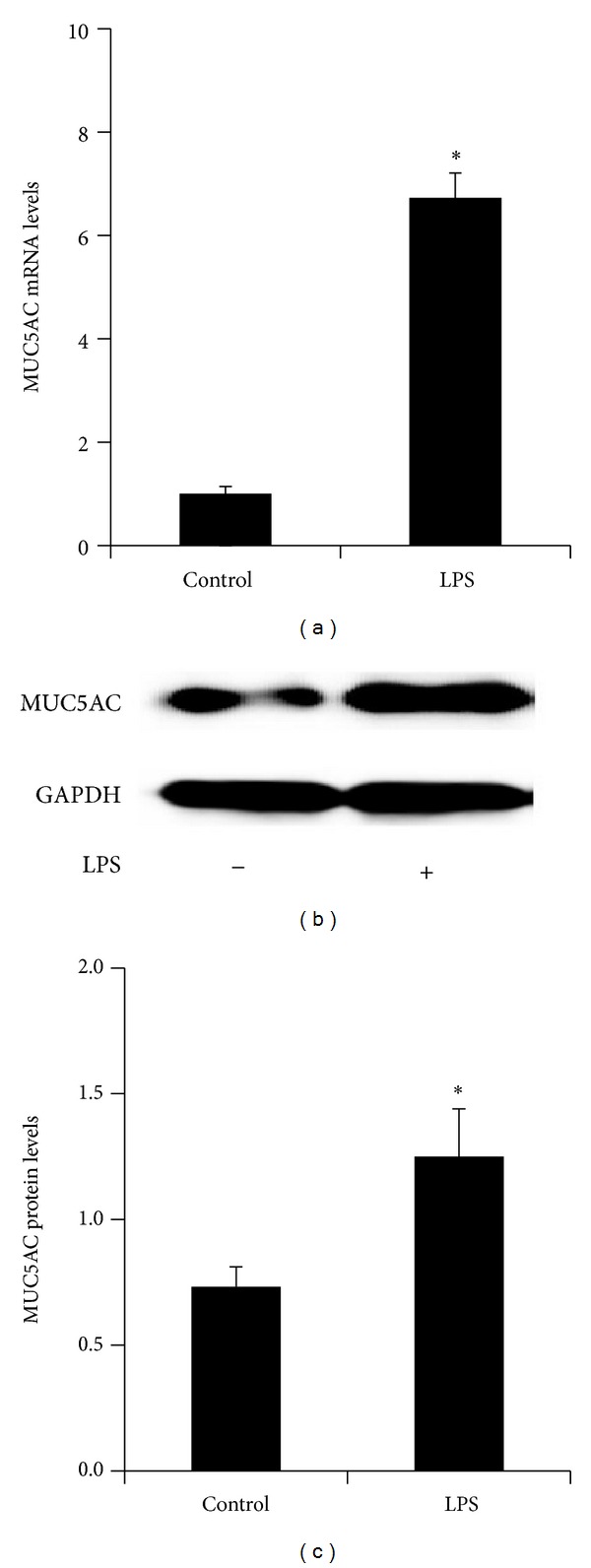
LPS upregulates MUC5AC expression in HIBECs. (a) Real-time PCR analysis showing that LPS increased MUC5AC mRNA levels in HIBECs. ((b), (c)) Western blot analysis showing that LPS increased MUC5AC protein levels. The mRNA or protein levels were measure by MUC5AC/*β*-actin. *P < 0.05.

Next, we validated the impact of LPS on MUC5AC expression in HIBECs. The real-time PCR analysis showed that the MUC5AC mRNA levels were significantly upregulated after LPS treatment (Figure 2(a)). The western blot analysis showed that MUC5AC protein levels were upregulated after LPS treatment in HIBECs (Figures 2(b) and 2(c)). Therefore, these data indicated that LPS increased MUC5AC expression in HIBECs.

### 3.3. LPS Increased MUC5AC Expression through Promoting EGFR Activation in HIBECs

**Figure 3:**
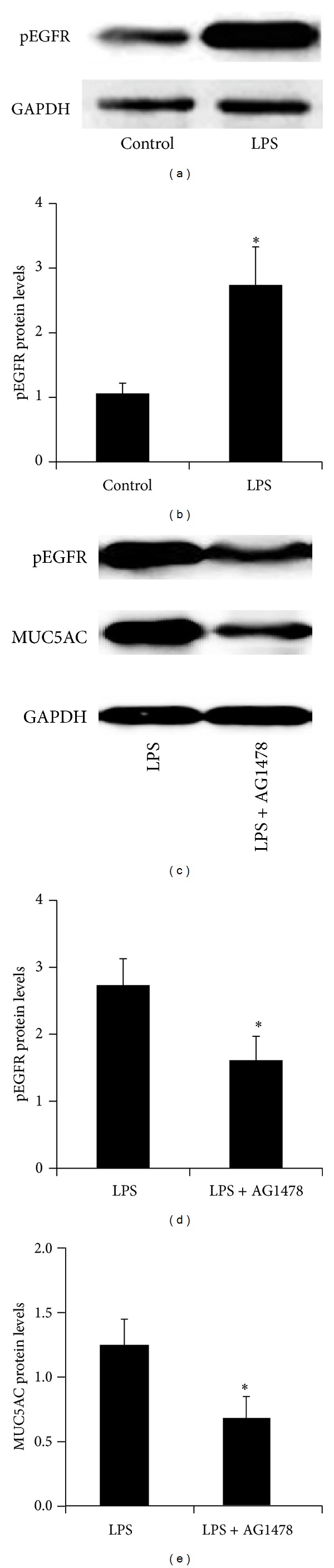
LPS increased MUC5AC expression through promoting EGFR activation in HIBECs. ((a), (b)) Western blot analysis showing that LPS increased pEGFR protein expression. ((c)–(e)) Western blot analysis showing that AG1478, an EGFR inhibitor, inhibited LPS-induced pEGFR and MUC5AC protein expression in HIBECs. Protein levels were measure by MUC5AC/*β*-actin. *P < 0.05.

**Figure 4:**
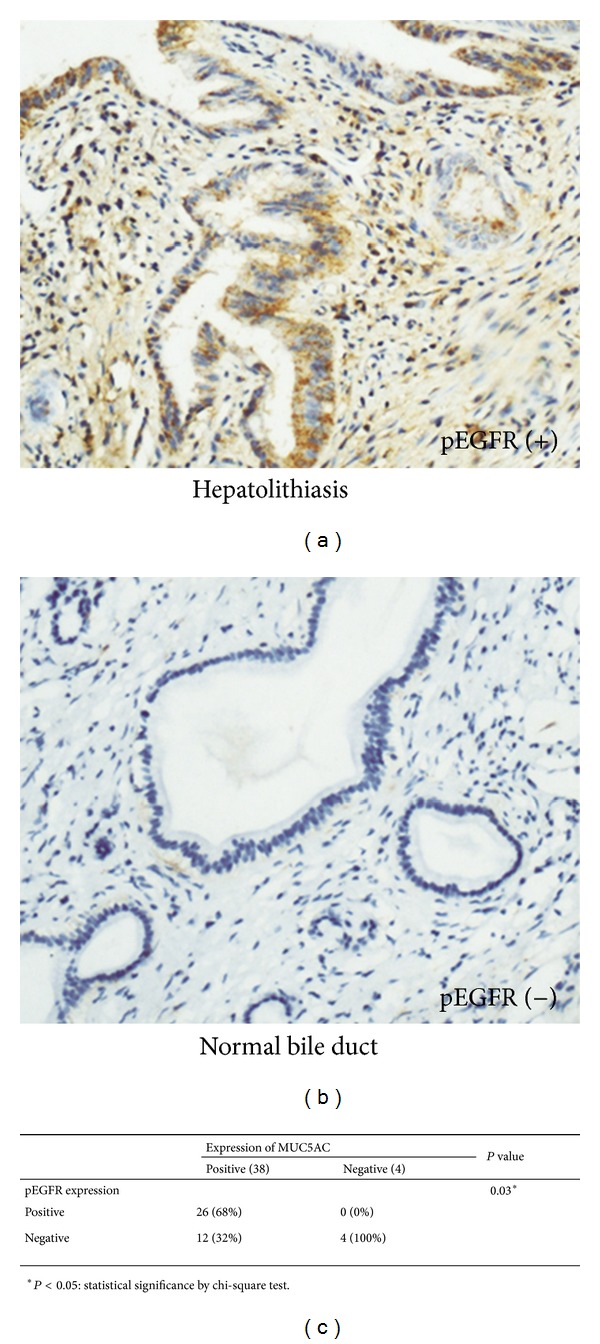
Expression of pEGFR correlated with MUC5AC expressions in 42 hepatolithiasis samples by IHC. (a) Positive staining of pEGFR in the hepatolithiasis sample. (b) Negative pEGFR staining in normal bile duct samples obtained from haemangioma patients. (c) Correlation between pEGFR and MUC5AC expression in 42 hepatolithiasis samples. *Statistical significance by chi-square test.

We then investigated the mechanism by which LPS increased MUC5AC expression in HIBECs. The western blot analysis revealed that phospho-EGFR (pEGFR) protein levels were significantly upregulated by LPS treatment in HIBECs (Figures 3(a) and 3(b)), indicating that EGFR was activated by LPS in HIBECs. Then HIBECs were treated with AG1478 to inhibit EGFR activation. The western blot analysis showed that LPS-induced EGFR phosphorylation was significantly inhibited by AG1478 in HIBECs (Figures 3(c) and 3(d)). Moreover, LPS-induced MUC5AC overexpression was significantly inhibited by AG1478-treatment in HIBECs (Figures 3(c) and 3(e)). Then we validated the correlation between MUC5AC and pEGFR in hepatolithiasis samples. 26 of the 42 (62%) hepatolithiasis samples showed positive pEGFR staining (Figure 4(a)). However, all 15 normal bile duct tissue samples showed pEGFR negative staining (Figure 4(b)). Expression of pEGFR significantly correlated with MUC5AC in hepatolithiasis samples (*P* = 0.03, Figure 4(c)). Together, the data suggested that LPS increased MUC5AC expression through promoting EGFR activation in HIBECs.

### 3.4. LPS Increased TGF-*α* Secretion in HIBECs

**Figure 5:**
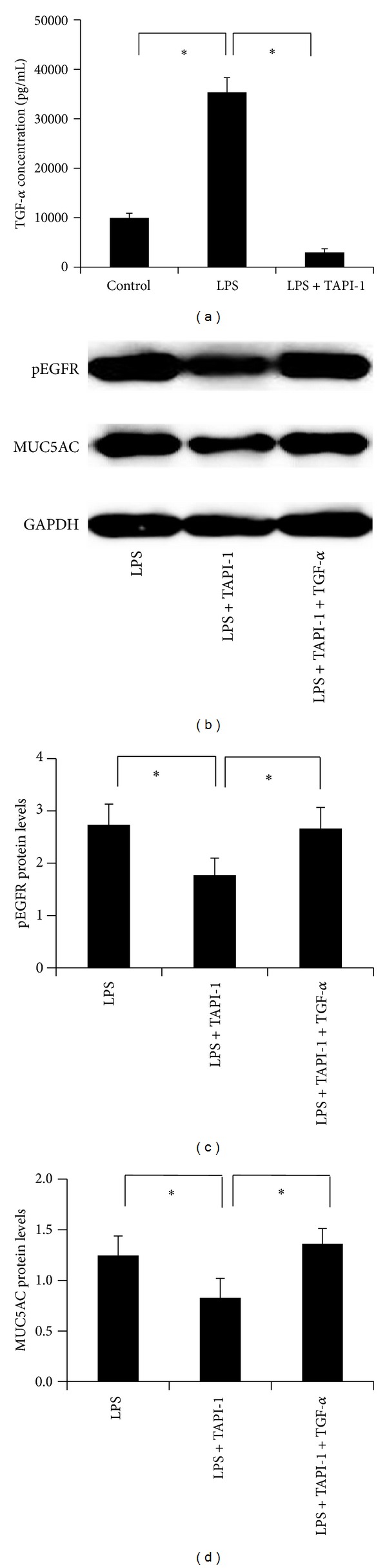
Inhibiting TACE blocked LPS-induced MUC5AC expression in HIBECs. (a) ELISA analysis showing that LPS increased TGF-*α* secretion in HIBECs, and this is inhibited by TAPI-1 treatment, a TACE inhibitor. ((b)–(d)) Western blot analysis showing that TAPI-1 inhibited LPS-induced pEGFR and MUC5AC protein expression. Protein levels were measure by MUC5AC/*β*-actin. *P < 0.05.

Then we investigated how LPS promoted EGFR activation in HIBECs. TGF-*α* is a putative ligand of EGFR. By ELISA analysis, we found that TGF-*α* secretion was significantly increased by LPS treatment in HIBECs (Figure 5(a)), indicating that LPS promoted EGFR activation by increasing TGF-*α* secretion in HIBECs.

### 3.5. TACE Activity Was Essential for LPS-Induced EGFR Activation and MUC5AC Overexpression in HIBECs

TACE is reported to plays an important role in TGF-*α* maturation and secretion; therefore, we used TAPI-1 to inhibit the TACE activity in HIBECs. The ELISA analysis showed that LPS-induced TGF-*α* secretion was significantly inhibited by TAPI-1 (Figure 5(a)), indicating that TACE activity is essential for LPS-induced TGF-*α* secretion in HIBECs. In addition, LPS-induced pEGFR expression was inhibited after TAPI-1 treatment (Figures 5(b) and 5(c)). Concomitantly, LPS-induced MUC5AC protein expression was also inhibited after TAPI-1 expression (Figures 5(b) and 5(d)). Moreover, exogenous supplement of TGF-*α* compensated the inhibitory role of TAPI-1 (Figures 5(c) and 5(d)). Together, our data suggested that TACE activity is essential for LPS-induced EGFR activation and MUC5AC overexpression in HIBECs.

## 4. Discussion

Hepatolithiasis is a multifactorial disease, and mucin overexpression, especially MUC5AC, plays important roles in its development. LPS, a main reason for hepatolithiasis, increases MUC5AC expression. In the present study we observed an overexpression of MUC5AC in the bile ducts of hepatolithiasis patients. The polymeric form and abundant O-linked oligosaccharides of MUC5AC increase the bile viscosity, which results in bile flow retardation and accelerates stone formation. Besides, mucin, especially gel-forming mucins such as MUC5AC, is the major component of biliary stones. It aggregates bile ingredients, such as bilirubin crystal and deciduous epithelium, which generate the core or lamellar backbone of calcium-bilirubinate stones [18, 19]. MUC5AC overexpression may also cause a decrease in the pH of bile, thereby promotes the lithogenic role of *β*-glucuronidase in the deconjugation of bilirubin diglucuronide [9]. Therefore, targeting MUC5AC overexpression might inhibit the above pathways and block stone formation and recurrence.

The regulatory pathway involved in MUC5AC overexpression is quite different in different cell types [20–22]. It has been reported that the EGFR cascade causes MUC5AC overproduction in gallbladder epithelial cells and promoting stone formation in cholecystolithiasis [11]. In the present study, we found that the EGFR pathway increased MUC5AC expression in HIBECs, which broadened the potential role of EGFR cascade in stone formation of biliary system. EGFR can be activated by several potent ligands. We found that LPS-induced TGF-*α* secretion activated EGFR and thus increased MUC5AC expression in HIBECs. TACE which is also known as a disintegrin and metalloprotease (ADAM-17), a member of the ADAM family of metalloproteases, is required in cell surface proteolytic processing of TGF-*α* in immortalized embryonic fibroblasts and primary keratinocytes [23]. Our results showed that TACE inhibitor significantly decreased TGF-*α* secretion in HIBECs. In addition, inhibiting TACE activity significantly abolished LPS-induced MUC5AC overexpression, indicating its pivotal role in LPS-induced MUC5AC overexpression in HIBECs.

An important purpose of investigating the pathogenesis of hepatolithiasis is to find the potential target to block the disease development. In the present study, we found that blocking EGFR and TACE both inhibited LPS-induced MUC5AC overexpression in HIBECs, indicating the potential target function of these two proteins. A recent study has reported that EGFR antagonist AG-1478 has a potent anti-MUC5AC overproduction effectiveness and holds promise as a candidate of hepatolithiasis [9]. Targeting TACE has been found to be a useful strategy in inflammation and EGFR-dependent tumours [13, 24]. In the present study, for the first time, our data indicated that targeting TACE might be a potential therapeutic strategy for inhibiting stone formation and recurrence. However, there is quite a lot of work needs to be done. The role of targeting EGFR or TACE should be investigated in more studies, especially in animal or clinical studies. Besides, between the two potential targets, which one has a better effects and less side reactions should also be studied. On this point, in our opinion, as EGFR participated in many physiological processes in biliary epithelial cells, inhibiting its upstream target TAEC seems to have less side reactions for therapy strategy.

There are limitations to this study, which require further investigation. The data obtained were from experiments based on HIBECs cultured in vitro; thus, as a more complex microenvironment in vivo, whether blocking TACE or EGFR inhibits MUC5AC overexpression in bile ducts in vivo requires further study. Among the putative ligands of EGFR, EGF is reported to play the important role in EGFR activation in gallbladder epithelial cells [11]. Thus, whether other potential ligands such as EGF play roles in EGFR activation in HIBECs needs to be studied further. Besides, the pathway between EGFR and MUC5AC in HIBECs is not very clear. It is reported that increased Ca^2+^ levels, extracellular signal-regulated kinase (ERK), and AKT is involved in the MUC5AC overexpression in human bronchial epithelial cells and rat conjunctival goblet cells [25, 26]. Our newly incomplete results indicated that LPS increased phospho-ERK (pERK) expression in HIBECs by western-blot analysis. Besides, blocking EGFR activation by AG1478 significantly inhibited LPS-induced pERK upregulation in HIBECs (data not shown). Based on these observations, we postulated that ERK might play a potential role in LPS-induced MUC5AC overexpression in HIBECs. However, it should be investigated in further studies.

In conclusion, these results suggest that LPS increases MUC5AC expression through the TACE/TGF-*α*/EGFR pathway in HIBECs. This new finding might give light to the prevention of stone formation and recurrence of hepatolithiasis.

## Figures and Tables

**Table 1:** Primer sequences used in the study.

	Sequences	Product length
MUC5AC	Forward: AACCGGCTGCTGCTACTCCTG	259
	Reverse: AGCGTGGGTGTAGGTGTGCAG	
GAPDH	Forward: ACCCATCACCATCTTCCAGGAG	159
	Reverse: GAAGGGGCGGAGATGATGAC	
